# Hot press sintering of Y_2_O_3_-Al_2_O_3_ glasses: Impact of particle size on thermal behaviour, sintering ability and mechanical properties of sintered bodies

**DOI:** 10.1016/j.heliyon.2024.e41260

**Published:** 2024-12-17

**Authors:** Melinda Majerová, Anna Prnová, Jana Valúchová, Monika Michálková, Beáta Pecušová, Michal Žitňan, Róbert Klement, Dušan Galusek

**Affiliations:** a Institute of Measurement Science V.V.I., Slovak Academy of Sciences, Dúbravská cesta 9, Bratislava, Slovakia; b Centre for functional and surface functionalized glass, Alexander Dubček University of Trenčín, Študentská 2, Trenčín, Slovakia; c Vitrum Laugaricio-Joint Glass Center of the IIC SAS, TnUD and FCHTP STU, Študentská 2, Trenčín, Slovakia

**Keywords:** Y_2_O_3_-Al_2_O_3_ glasses, Eutectic composition, Milling, Thermal properties

## Abstract

The impact of grinding on particle size, thermal behaviour, and sintering ability of yttrium aluminate glass microspheres with eutectic composition (76.8 mol % Al_2_O_3_ and 23.2 mol % Y_2_O_3_) was studied. The work was conducted with the aim of determining the optimal particle size and grinding conditions of glassy powder used for hot-pressing of ceramic and glass-ceramic materials with desired mechanical properties. The flame synthesis was used for the preparation of glass microspheres (diameter ∼ 40 μm). Ball milling procedure under different conditions was applied to adjust of size of prepared microbodies. The milled powders were subsequently hot pressed. The prepared samples (raw, milled microspheres and hot-press sintered bodies were characterised by X-ray powder diffraction, and scanning electron microscopy. Thermal analysis and particle size analysis in combination with X-ray powder diffraction and high temperature X-ray diffraction was used for detailed inspection of thermal behaviour and phase changes in raw and milled systems in the temperature interval 25–1200 °C. The samples after flame synthesis and after milling were found to be X-ray amorphous. The particle size measurements showed that the systems with a smaller average size D [0.9] ∼ 25 μm and a monomodal particle size distribution were prepared after 6h of milling. Thermal analysis in combination with X-ray and high temperature X-ray analysis indicated a difference in the crystallization mechanism of the yttrium aluminate garnet phase depending on the milling time. The bulk glass ceramic with fine lamellar eutectic microstructure and interesting mechanical properties (Vickers hardness H_V=_17.6 ± 0.2 GPa, indentation fracture toughness K_IC_ = 4.3 ± 0.3 MPa.m^1/2^) resulted from the sintering of microspheres milled for 6h under the “gentlest” conditions (milling speed - 200 rpm, and milling balls size - 5 mm in diameter).

## 1 Introduction

Ceramics with eutectic microstructures are a suitable replacement for polycrystalline yttrium aluminate garnet (YAG), especially for high-temperature applications. Generally, oxide eutectic ceramics have a high melting temperature, excellent thermal stability, high oxidation resistance, and desired mechanical properties not only at room temperature but also at elevated temperatures, due to the strong eutectic interface between different phases [1,2]. Due to the afore mentioned properties, eutectic ceramic materials are actively being researched for applications in areas such as aeronautics, aerospace, and high-efficiency power generator systems. These materials are one of the most promising materials for ultra-high temperature structural materials applied to the high temperature oxidizing atmosphere over a long period of time [[3], [4], [5]]. Eutectic microstructures are also difficult to prepare and require expensive and technically demanding equipment, so far facilitating the fabrication of only relatively small pieces. The preparation and properties of eutectic systems were studied in single crystals (Al_2_O_3_ and Y_3_Al_5_O_12_ phases in the whole piece of the material have only one growth orientation for each phase) but also as polycrystalline systems [6,7]. Eutectic single crystals were prepared by various directional solidification techniques, such as laser floating zone technique, optical floating zone technique, edge-defined film-growth technique, seeding technique, micro-pulling-down technique, and Bridgman technique [6,[8], [9], [10], [11]]. However, the use of single crystals is limited by their size, which is determined by the conditions of their preparation. Polycrystalline eutectic materials can be prepared e.g. by solid-state reaction route, pressing and subsequent thermal annealing, hot-press method [[12], [13], [14]]. Al_2_O_3_ and Y_3_Al_5_O_12_ polycrystalline eutectics were prepared by hot-pressing from arc-melted pellets: after the melting the pellets were crushed, ground and sieved through 150 μm sieve [14]. The prepared samples showed an average flexural strength 274 ± 61 MPa. Zhang et al. studied the mechanical properties of Al_2_O_3_/YAG binary eutectic and Al_2_O_3_/YAG/ZrO_2_ ternary eutectic ceramics prepared by laser melting [15]. The studied system had Vickers hardness 16.7 ± 2 GPa for Al_2_O_3_/YAG/ZrO_2_ system and 17.5 ± 2 GPa for Al_2_O_3_/YAG. It follows that the mechanical properties of eutectic systems can be significantly influenced by the preparation conditions as well as the composition.

Another option for the preparation of the eutectic system with the composition Al_2_O_3_-Y_3_Al_5_O_12_ is the combination of hot press synthesis and controlled crystallization of amorphous glass, during which the mechanical properties of glass are improved [16]. In this case, first, preparing the glasses with yttria-alumina eutectic composition is necessary. In general, the preparation of aluminate glasses as well as yttrium-aluminate glasses is a complex process since these glasses have high melting temperatures and a high tendency to crystallization. For their preparation, containerless melting methods are used to eliminate heterogeneous nucleation on the surfaces of the melting container, thereby suppressing crystallization during cooling (e.g. melting in aero-acoustic levitator, (AAL) or conical-nozzle levitator (CNL)) [[17], [18], [19]]. In 2004, Roseflanz et al. described flame synthesis as another possibility for the preparation of difficult-to-melt glasses [20]. The products of flame synthesis are glass microspheres. Bulk glasses can be prepared from glass microspheres by hot-pressing. However, the systems prepared by flame synthesis have a wide particle size distribution ranging from 1 μm to 150 μm. To achieve the desired results, the particle size distribution must be adjusted. Milling appears to be a possible solution. The process would also eliminate (crush) hollow microspheres, which act as defects in the microstructure of sintered bodies. To correctly select the conditions of HP experiments and to achieve the desired phase composition by controlled crystallization, the thermal behavior of such modified systems must be examined.

The effect of particle size on the mechanical properties of eutectic systems with different compositions have been studied by many groups [13,16,21]. Isobe et al. fabricated an Al_2_O_3_-GdAlO_3_ eutectic composite by spark plasma sintering of a powder prepared by induction-melting [13]. To monitor the effect of particle size on the microstructure and mechanical properties, the two fractions of the powder were prepared by sieving (3–44 μm, 64–124 μm). While the particle size did not significantly affect the Vickers hardness (approx. 19.0 GPa in the case of both factions) the flexural strength was significantly higher in the case of the finer powders 3–44 μm (605 MPa) than in the coarser powders 64–124 μm (402 MPa). The dependence between grain size and hardness of Al_2_O_3_-Y_2_O_3_-ZrO_2_ eutectic system prepared by HP was also studied [21]. Microcomposite with the grain size of 0.5–1.8 μm, had the highest Vickers hardness H_V_ = 19 GPa.

The effect of the method of preparation of yttrium aluminate glasses on particle size, as well as the effect of the preparation method of bulk glasses of yttria-alumina eutectic composition on their mechanical properties, was studied by Prnová et al. [16]. The eutectic glassy precursors materials were prepared by sol-gel Pechini method and flame synthesis. The particle size of precursor materials was modified by a vibratory micro mill. The results of the Particle Size Analysis (PSA) conducted on the prepared systems revealed insightful findings regarding the impact of grinding time on particle size and distribution. After 9 h of intense grinding, there was a notable decrease in particle size, achieving uniform, the monomodal distribution within the system. This optimal particle size was favourable for further processing. However, extending the grinding time to 12–15 h led to additional particle size reduction, and surprisingly to the bimodal distribution. This shift in particle distribution characteristics was deemed undesirable, particularly for the subsequent sintering process. Three methods for preparation of bulk samples were applied: hot pressing (HP), rapid hot pressing (RHP), and spark plasma sintering (SPS). The sample prepared by the HP method from the precursor system with median particle size of 8.6 μm had the best mechanical properties (H_V_ = 17 GPa and K_IC_ = 4.2 MPa.m^1/2^).

In general, literature indicates that ball milling is utilized in two primary types of processes. The first involves milling a mixture of powders, comprising various metals, alloys, and compounds, to achieve a homogeneous blend. This method is known as mechanical alloying, a typical solid-state process [22,23]. Through mechanical alloying, a variety of alloys can be produced, including nickel-based, aluminum-based, magnesium-based, and high-entropy alloys [[24], [25], [26], [27], [28]]. In the second type of process, powders with a consistent composition are milled without any material transfer taking place. This is referred to as mechanical milling. To obtain the desired products, several factors must be considered, such as the milling atmosphere, ball-to-powder ratio, milling speed, ball size, milling duration, and the initial size range of the powders [29,30]. Erdemir's research [30] has shown that in the case of high-energy ball milling of B_4_C powders, the key parameters influencing particle size reduction are milling speed and milling time.

This work aimed to optimize the grinding of glass microspheres after flame synthesis (FS) to achieve fine eutectic microstructure of sintered bodies with improved mechanical properties. Investigating the effect of milling conditions on particle size and particle size distribution and the impact of these parameters on thermal behaviour and the sintering ability of milled systems was desirable for accomplishing this aim. The Pechini sol-gel method and flame synthesis (FS) were combined to prepare glassy powder with the eutectic composition (76.8 mol % Al_2_O_3_ and 23.2 mol % Y_2_O_3_). The prepared system was characterized by XRD, SEM and DTA/TG. Three different grinding speeds and grinding bodies with 5 and 11 mm diameter were selected, while individual grinding times did not exceed 9 h. Particle size and particle size distribution were verified by particle size analysis (PSA). DTA/TG, XRD, SEM and HT XRD analysis were used to obtain information about the thermal behavior of milled systems. Selected glassy powders were used for HP experiments to examine their sintering ability. The effect of ball milling on sintering ability and final properties of sintered bodies e.g. density, microstructure, and mechanical properties (Vickers hardness and indentation fracture toughness) were studied, and the results were discussed.

## 2 Experimental part

### 2.1 Glass microspheres preparation

The precursor powder for flame synthesis was prepared by sol-gel Pechini method [31] using Al (NO_3_)_3_.9H_2_O (99.9 %, Sigma Aldrich, Germany) and Y_2_O_3_ (99.9 %, Treibacher Industry, Austria). Citric acid (CA) and ethylene glycol (EG) were used as cross-linking agents. A detailed description of the preparation process is given in Ref. [32]. A narrow fraction of the precursor powder obtained by sieving through a 25 and 40 μm sieve was used for the flame synthesis. The powders were fed into methane-oxygen flame where the particles were melted and then quenched by deionized water. The glassy particles were collected in a stainless-steel vessel and separated by filtration through a ceramic filter. Carbon residua from the resulting glassy particles were eliminated by heating at the temperature of 650 °C for 4 h in ambient air.

### 2.2 Milling of microspheres

Three different milling experiments were performed using Retsch centrifugal ball mill S 100 (dry grinding; agate mortar and grinding balls). Based on the literature study [[22], [23], [24], [25], [26], [27], [28]] and the results of our previous work [16], we used gentle conditions (milling speed 200 rpm and smaller milling balls, ∅ = 5 mm) and more harsh grinding conditions (milling speed 350 rpm in combination with smaller (∅ = 5 mm), medium (∅ = 11 mm) and large (∅ = 20 mm) milling balls). The conditions for individual milling experiments (series) are summarized in Table 1**.** The weight ratio of grinding balls to powder was 3:1. Samples for particle size analysis were taken every hour until the total milling time of 9 h was achieved.

**Table 1:** The review of grinding conditions used in individual experiments.

Milling	Milling speed [rpm]	Diameter of milling balls [mm]
1. Series (S1)	200	5
2. Series (S2)	350	11
3. Series (S3)	350	20

**HP experiments:** Laboratory hot-press Classic 0220 ZL was used for the HP experiments. Approximately 1g of sample was placed into a graphite die with the inner diameter of 12 mm coated with boron nitride. The heating rate of 20 °C.min^−1^ was used to achieve the temperature of 1600 °C. The pressure of 30 GPa was applied at the beginning of each experiment. Dwell time 0 min was used to prevent grain growth during sintering. The final thickness of HP sintered samples was about 1.5 mm.

### 2.3 Characterisation

Scanning electron microscopy (SEM) was used to examine the morphology of microspheres and the microstructure of the HP sintered samples. The microspheres were fixed on a graphite tape. Sintered samples were cut, embedded in a polymer resin, carbon sputtered to prevent charging and examined be SEM (JEOL 7600F) at the accelerating voltage of 20 kV.

The NIS Elements AR 4.30.00 software was used to calculate the size of the dark areas in the SEM images of the sintered samples microstructure, corresponding to the Al_2_O_3_ phase. For this purpose, five images of each sample were taken at 5000× magnification. A minimum of 80 particles were manually drawn on each photo (i.e. for each sample at least 400 particles) and the data was evaluated.

Particle size analysis (Mastersizer 2000, Malvern Panalytical) was carried out to obtain information about particle size distribution. Approximately 0.18 g of microspheres were dispersed in 40 mL of water solution of sodium pyrophosphate (concentration = 0.15 g/L). The resulting mixture was dispersed in an ultrasonic bath for 5 min. Before measurement, the dispersed solution was diluted with deionized water to a volume of 300 mL, and 4.8 mL of sodium pyrophosphate was added into the resulting mixture.

The DTA/TG analysis in the temperature interval 25–1200 °C was performed using Netzsch STA 449 F1 Jupiter analyser. About ∼100 mg of microspheres was placed into a corundum crucible and heated at a heating rate of 10 °C.min^−1^ in N_2_ atmosphere. Empty corundum crucible was used as a reference. A correction on empty sample crucible was performed before each analysis to obtain reliable data. The obtained data were processed using the Netzsch Proteus Thermal Analysis Version 6.0.0 software.

Phase analysis of raw and milled microspheres and the samples after DTA was performed using powder X-ray diffraction (Panalytical Empyrean, CuKα radiation with a wavelength of 1.5405 Å). The XRD patterns were recorded in the 2Θ range 10–80°.

The high temperature XRD (HT XRD) analysis was performed using X-ray device (DY1098, Panalytical Empyrean) equipped with a high-temperature cell Anton Paar HTK 16. A suspension of powder in isopropyl alcohol was prepared using ∼30 mg of each sample. The suspension was deposited in a thin uniform layer on a Pt/Rh heating strip that served also as a sample holder during the measurement. Precision temperature control during analysis was provided by a Pt/Pt/Rh 10 (S-type) thermocouple welded to the bottom of the Pt/Rh strip. First, two long scans (step 0.05 °.s^−1^, accumulation time 194 s per step) of the sample were measured from 10 to 80° in 2 theta range at a laboratory temperature (25 °C) and at 750 °C. Subsequently, the sample was heated at a rate of 5 °C.min^−1^ and every 5 °C in the temperature interval of 750–1450 °C a short scan was measured. The short scan was measured with the step of 0.05 °.s^−1^ and the accumulation time of 14 s per step in the 2θ range of 30–60°. After the last short scan at 1450 °C, the sample was quickly cooled to 25 °C and another long scan was recorded.

All measured diffraction patterns were evaluated using the software High Score Plus (v.3.0.4, PAN Analytical, The Netherlands) with the use of COD2019 and the PDF4 database.

Density measurements of raw microspheres and HP sintered bodies were performed using He-pycnometer (Quantachrome Ultrapyc 1200). A small measuring cell and a sample weight ⁓1 g were used in the case of powdered samples. For sintered bodies, the weight of the samples was around 1g.

The Vickers hardness (H_V_) and indentation fracture toughness (K_IC_) measurements were obtained as a mean value from 10 measurements. Each measurement was performed on a polished sample using a Micro Hardness Tester WIKI 200, with the indentation load force of 9.8 N (1 kgf) using observation objective with 500 × magnification and a high-resolution camera with auto focus and auto reading function. The dwell time of indentation was 10 s. The H_V_ value was calculated from auto-measurement of the indent size. The indentation fracture toughness was calculated from the length of radial cracks created in the corners of imprints after indentation by the method described by Anstis [33].

## 3 Results

### 3.1 Basic characterisation of glasses

**Fig. 1:**
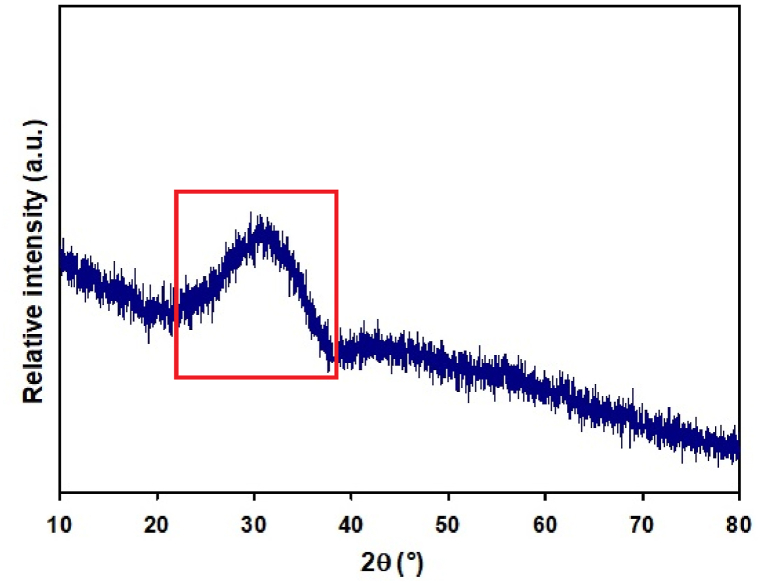
XRD patterns of glass microspheres after flame synthesis, the broad shoulder (indicating amorphous character) is signed by red color.

The glassy powder prepared by the flame synthesis was examined by XRD to confirm its amorphous nature. The XRD pattern (Fig. 1) shows only the presence of a broad shoulder in the 2θ interval 25–40°. This shoulder is specific for X-ray pattern of glasses and amorphous materials [34,35]. No discrete diffractions which would suggest the presence of crystalline phases were observed in the XRD pattern. If the sample contained a small number of crystalline phases, the content was below the detection limit of X-ray diffraction.

**Fig. 2:**
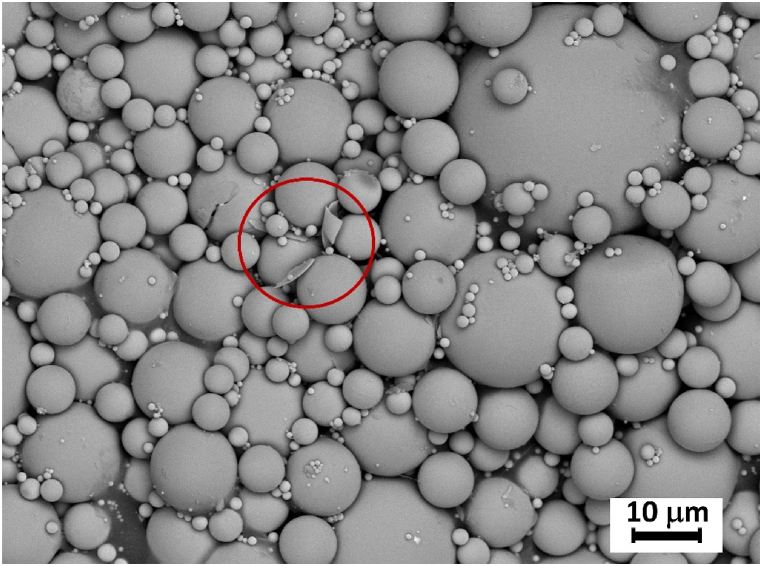
SEM micrograph of glass microspheres after FS, the fragments of the hollow sphere are marked by the red circle.

**Fig. 3:**
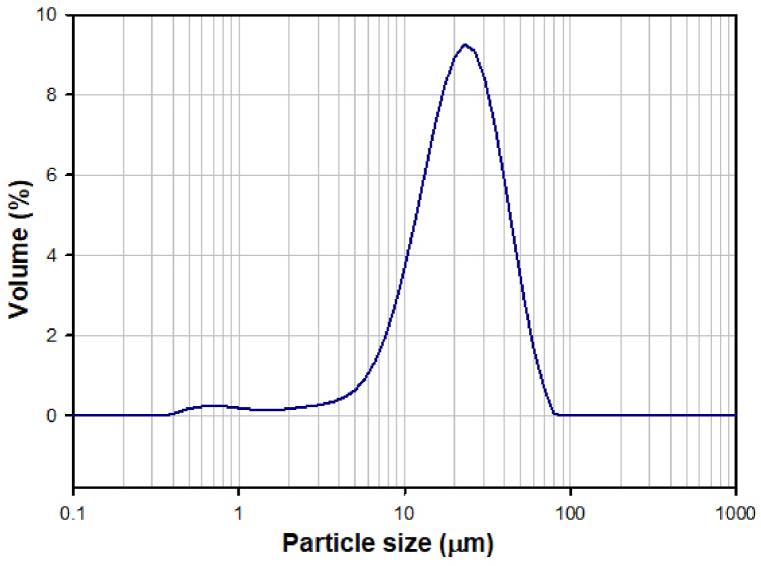
Particle size distribution of microspheres after FS.

Subsequent detailed inspection of the morphology of prepared particles by SEM showed the presence of fully remelted, spherical particles with smooth surface (Fig. 2). No crystalline or partially crystalline particles were observed, but a small number of hollow microspheres was observed, which resulted from the presence of a small amount of residual carbon in the precursor powders. Particle size analysis (Fig. 3) showed a bimodal distribution of the particles, with the diameters ranging from 1 to 60 μm, while 90 vol% of sample (D (0.9) parameter) is represented by particles with the size ≤40 μm.

### 3.2 Milling experiments and results of PSA analysis

**Fig. 4:**
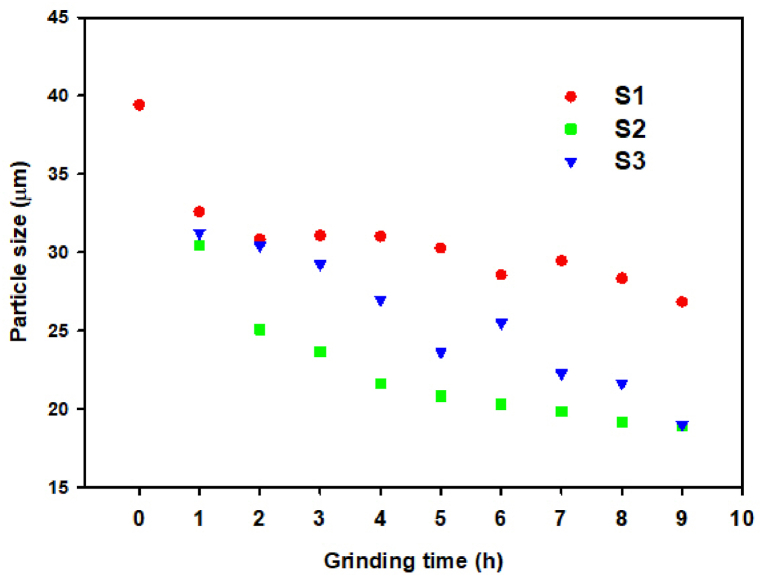
The change of particle size (D (0.9) parameter) in individual experiments.

**Fig. 5:**
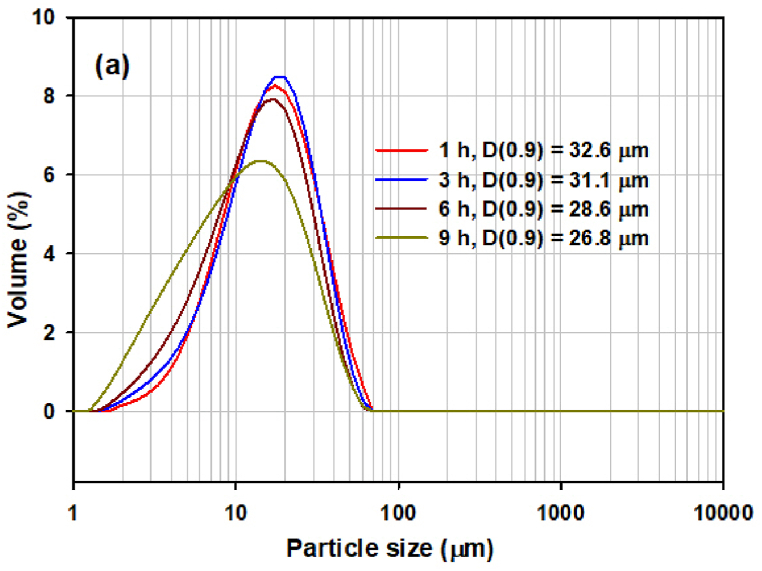

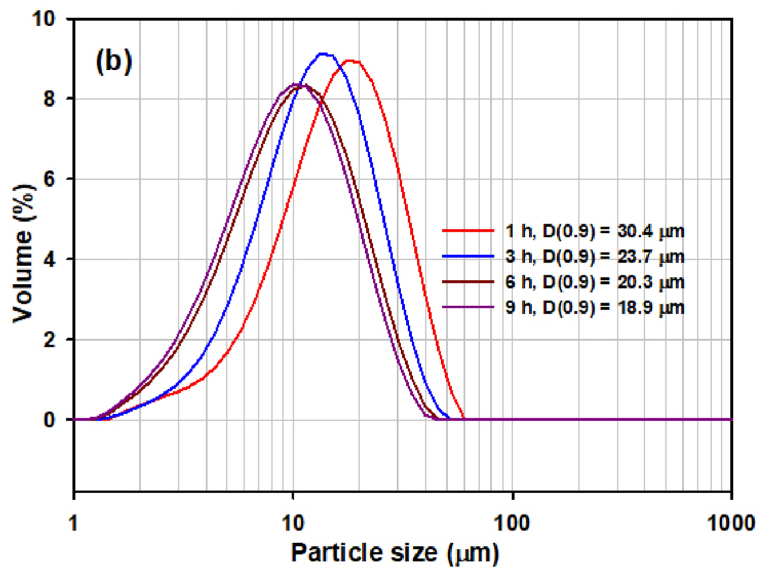

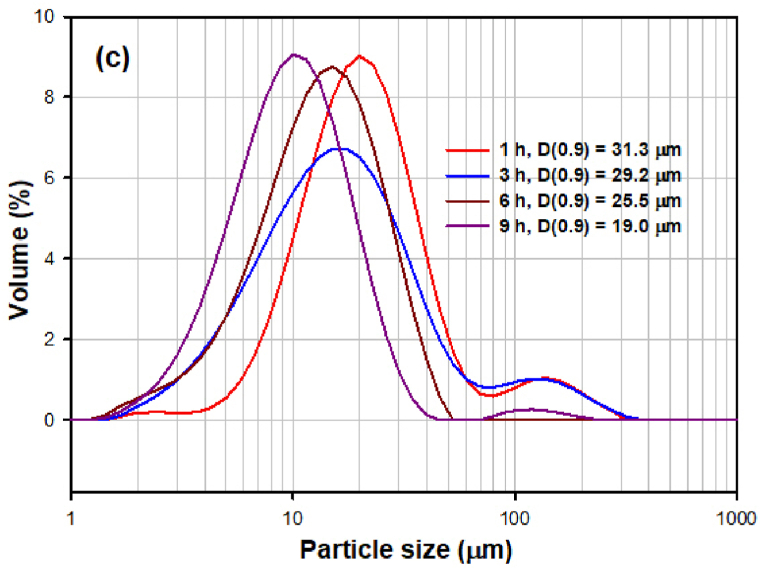

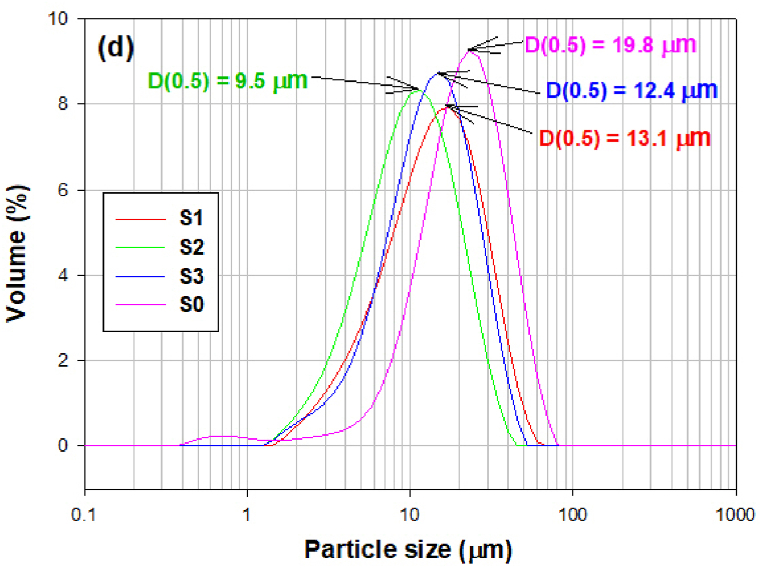
(a) The PSD of samples milled for 1, 3, 6 and 9 h in the milling experiment S1. (b) The PSD of samples milled for 1, 3, 6 and 9 h in the milling experiment S2. (c) The PSD of samples milled for 1, 3, 6 and 9 h in the milling experiment S3. (d) The PSD of samples milled for 6 h under different conditions (milling experiment S1, S2, S3) and raw microspheres (S0, without milling).

Based on the results of our previous work [16], three grinding experiments were proposed with the aim of reducing the grinding time and optimizing the particle size in the powders intended for HP (Table 1). These differed by the size of the used milling bodies and the milling speed. The effect of milling on particle size and particle size distribution was studied. The samples in individual experiments were milled for 9 h, and after each hour of milling PSA were performed. The results are shown on Fig. 4. The most significant reduction in particle size under all milling conditions occurred already after 1 h of milling. The PSD of the samples measured after 1, 3, 6 h of the milling and at the end of the experiment is shown on Fig. 5a, 5b and 5c. In the first experiment (Fig. 5a), the curves measured after 1, 3 and 6 h of milling show monomodal particle size distributions with a wide distribution interval (span value ranged from 1.697 to 1.825), with the particle size (D (0.9) parameter) ranging from 31.7 μm to 28.5 μm. However, after 9 h of milling, the distribution interval begins to widen (span value 2.283), indicating a lower uniformity of particles in the studied system.

Fig. 5b shows the PSD curves obtained after 1, 3, 6 and 9 h in the milling experiment S2. The relatively narrow monomodal distribution with a wide distribution interval (span = 1.580) was obtained after 3 h of milling. After 6 and 9 h of milling, some widening of the interval (span = 1.740–1.749) was observed, but this was not as pronounced as after 9 h of milling in experiment S1.

Fig. 5c shows the comparison of PSD of samples milled for 3, 6 and for 9 h in the experiment S3. Trimodal (after 1 h) and bimodal PSD (after 3, 6 and 9 h) is observed in the samples, demonstrated as the shoulders in the particle size intervals 1.5–4 μm and 60–120 μm. The presence of the first shoulder is caused by the formation of very small particles during more vigorous milling. The presence of the shoulder in the interval 60–120 μm results from the formation of agglomerates in the system.

By comparing particle sizes, widths of distribution intervals and PSD curves after individual experiments, it can be concluded that a relatively narrow distribution and satisfactory particle size was achieved in all samples after 6 h of grinding (Fig. 5d).

The values of the individual parameters in the samples after 6 h of milling are summarized in Table 2. The lowest particle size (from 3.76 μm to 20.31 μm) was achieved when using grinding bodies with a diameter of 11 mm and the milling speed of 350 rpm. The particle size from 4.86 to 25.5 μm was obtained when grinding bodies with a diameter of 20 mm and the same milling speed as in the previous experiment were used. The lowest grinding efficiency in terms of particle size was observed in the first experiment (milling speed 200 rpm and the diameter of grinding bodies 5 mm).

**Table 2:** Results of PSA of raw microspheres (S0) and samples milled for 6h.

Sample	Series of grinding	D(0.1)	D(0.5)	D(0.9)	SPAN
S0	–	8.1	19.8	39.4	1.576
S1	1	4.6	13.1	28.6	1.825
S2	2	3.8	9.5	20.3	1.749
S3	3	4.9	12.4	25.5	1.661

**Fig. 6:**
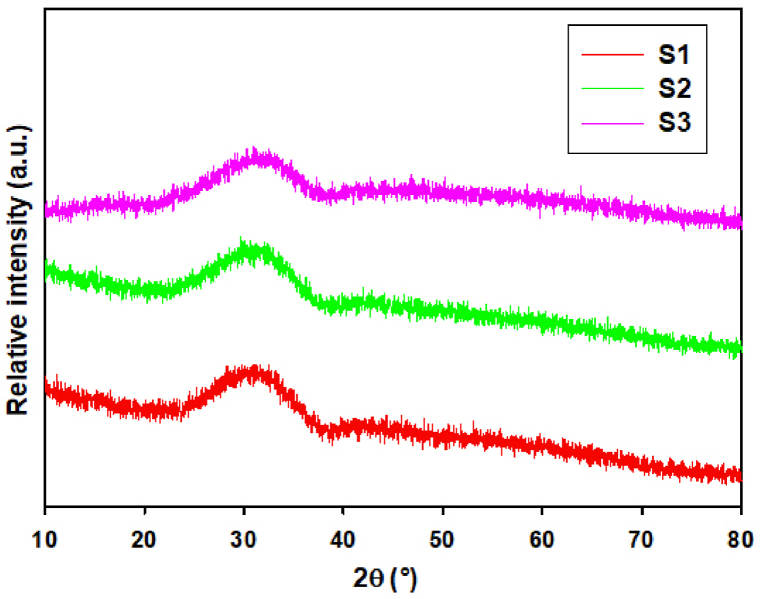
The X-ray patterns of samples milled for 6h.

After milling all samples were subjected to XRD analysis (Fig. 6). The results confirmed that all milled samples were X-ray amorphous. No traces of any crystalline phases (YAG, Al_2_O_3_) that could be expected in this system were observed in the samples and the used milling conditions did not cause any mechano-activation in the system.

**Fig. 7:**
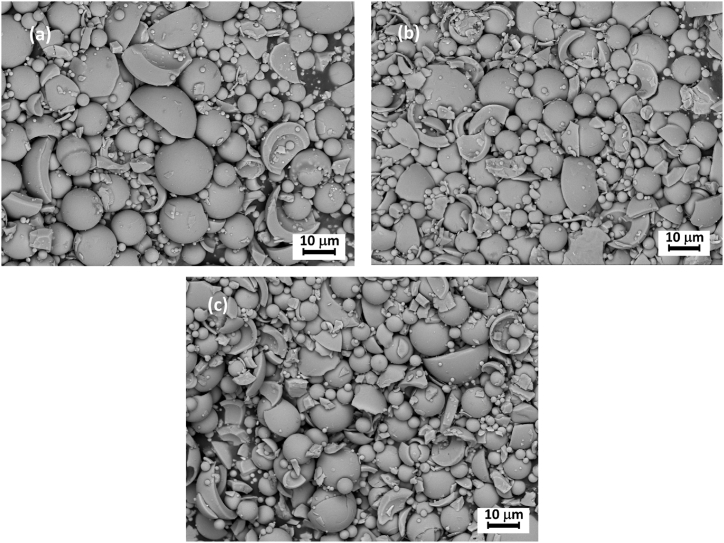
SEM images of YA glass samples milled for 6 h (a) S1 (b) S2 (c) S3 TA, HT XRD and XRD of milled systems.

Fig. 7 shows the SEM micrographs of the microspheres milled for 6 h. The fragments of crushed particles are visible, consisting mainly of the broken shells of hollow microspheres.

Samples milled from 1 to 9 h were selected to study their thermal behavior. Thermal analyses in the temperature interval 25–1200 °C were performed in nitrogen atmosphere to study physical changes in the systems. Also, to obtain information about phase changes in given temperature interval, all samples after TA (thermal analysis) were subjected to XRD analysis. HT XRD experiments of samples milled for 6 h were also performed.

**Fig. 8:**
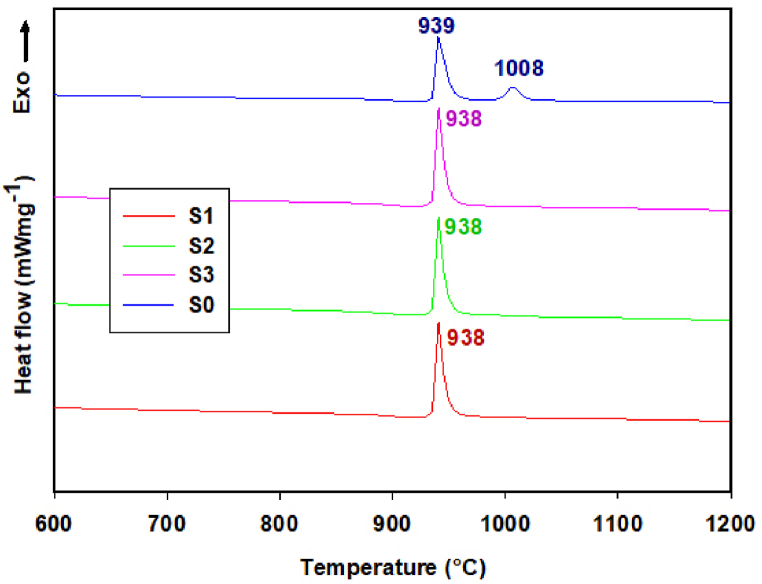
The comparison of DTA curves of the microspheres (S0) after FS and microspheres milled for 6 h under different conditions (S1, S2, S3).

DTA curve of raw microspheres (after FS) shows two exothermic effects (Fig. 8), with the maximum at 938 and 1008 °C.

Similar thermal behavior and the presence of two exothermic effects were also observed in the samples milled for 1–5 h (results not shown). A significant change in the thermal behavior was observed in the samples milled from 6 to 9 h, where only one exothermic effect (at 938 °C) was present in the DTA records. The comparison of DTA curves of the systems milled for 6 h and the DTA curve of raw microspheres is shown in Fig. 8.

The characteristic temperatures (T_x_ - temperature of onset of exothermic effect, T_p_ - temperature of the maximum of exothermic effect, T_e_ - temperature of the end of exothermic effect) were determined from the DTA records, and the areas of exothermic effects were calculated. The results are presented in Table 3, Table 4, Table 5. The first 5 h of milling had no significant effect on the thermal behavior of the systems in the temperature interval 25–990 °C. In the case of the first exothermic effect, the difference in the values of characteristic temperatures was within the range of accuracy of the measuring device (±3 °C).

**Table 3:** The values of characteristic temperatures, determined from DTA curves of milled samples in milling experiment S1 (T_x_-temperature of the onset of exothermic effect, T_p_ – temperature of the maximum of exothermic effect, T_e_- temperature of the end of exothermic effect).

Sample name	T_x1_/°C	T_p1_/°C	T_e1_/°C	Area_1_/μVs/mg	T_x2_/°C	T_p2_/°C	T_e2_/°C	Area_2_/μVs/mg
**S1_0h**	930	939	949	34	1001	1008	1013	31
**S1_1h**	937	941	951	34	1003	1008	1019	29
**S1_2h**	937	941	950	35	1002	1007	1018	30
**S1_3h**	937	941	951	34	1001	1007	1018	29
**S1_4h**	936	940	950	35	1000	1006	1017	29
**S1_5h**	936	941	951	30	1000	1007	1018	24
**S1_6h**	936	938	941	60	–	–	–	–
**S1_7h**	936	938	941	60	–	–	–	–
**S1_8h**	937	939	942	62	–	–	–	–
**S1_9h**	936	938	941	60	–	–	–	–

**Table 4:** The values of characteristic temperatures, determined from DTA curves of milled samples in milling experiment S2 (T_x_-temperature of the onset of exothermic effect, T_p_ – temperature of the maximum of exothermic effect, T_e_- temperature of the end of exothermic effect).

Sample name	T_x1_/°C	T_p1_/°C	T_e1_/°C	Area_1_/μVs/mg	T_x2_/°C	T_p2_/°C	T_e2_/°C	Area_2_/μVs/mg
**S2_0h**	930	939	949	34	1001	1008	1013	31
**S2_1h**	936	941	950	35	1002	1007	1017	30
**S2_2h**	936	941	950	32	1000	1007	1017	26
**S2_3h**	936	941	951	34	989	1006	1018	26
**S2_4h**	936	940	950	31	997	1006	1017	24
**S2_5h**	937	941	950	34	997	1006	1017	26
**S2_6h**	937	939	941	60	–	–	–	–
**S2_7h**	937	939	941	60	–	–	–	–
**S2_8h**	937	939	941	61	–	–	–	–
**S2_9h**	937	939	941	61	–	–	–	–

**Table 5:** The values of characteristic temperatures, determined from DTA curves of milled samples in milling experiment S3 (T_x_-temperature of the onset of exothermic effect, T_p_ – temperature of the maximum of exothermic effect, T_e_- temperature of the end of exothermic effect).

Sample name	T_x1_/°C	T_p1_/°C	T_e1_/°C	Area_1_/μVs/mg	T_x2_/°C	T_p2_/°C	T_e2_/°C	Area_2_/μVs/mg
**S3_0h**	930	939	949	34	1001	1008	1013	31
**S3_1h**	935	944	957	39	999	1010	1019	32
**S3_2h**	937	942	952	35	997	1006	1017	26
**S3_3h**	937	941	950	35	995	1005	1016	24
**S3_4h**	937	941	950	36	995	1005	1016	23
**S3_5h**	935	945	959	40	998	1010	1020	24
**S3_6h**	937	939	941	61	–	–	–	–
**S3_7h**	937	939	941	60	–	–	–	–
**S3_8h**	937	939	941	61	–	–	–	–
**S3_9h**	937	939	941	61	–	–	–	–

In the case of the second exothermic effect (temperature interval 990–1200 °C), only a slight reduction of the characteristic temperatures (2–7 °C) with increasing milling time was observed, together with the narrowing of the crystallization interval, and acceleration of the second step of crystallization. The areas of both effects did not change significantly.

DTA records of the samples milled for 6–9 h show only one exothermic effect and the difference between T_x_ and T_e_ is only ∼ 5 °C, which means that the respective events take place in a narrower temperature interval and in a shorter time.

**Fig. 9:**
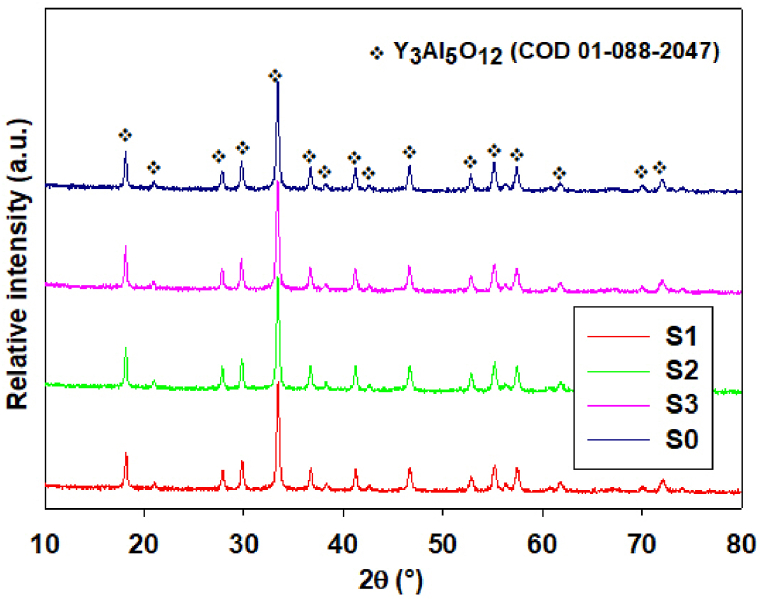
The comparison of X-ray diffraction patterns of the microspheres after DTA, S0 - raw microspheres (after FS) and microspheres milled for 6 h under different conditions (S1, S2, S3).

XRD analysis confirmed the presence of only YAG phase in all milled samples after DTA, which indicated crystallization of this phase in one or two steps depending on the milling time. The comparison of XRD patterns of the samples milled for 6 h with the XRD pattern recorded after DTA analysis of the raw microspheres (before milling) is shown in Fig. 9. Subsequently, the effect of milling on the amount of the emerging YAG phase in the samples was monitored. The comparison of the XRD patterns showed that the content of the YAG phase in the samples after DTA increases as the milling time increases, which could indicate a higher tendency of milled systems to crystallization.

**Fig. 10:**
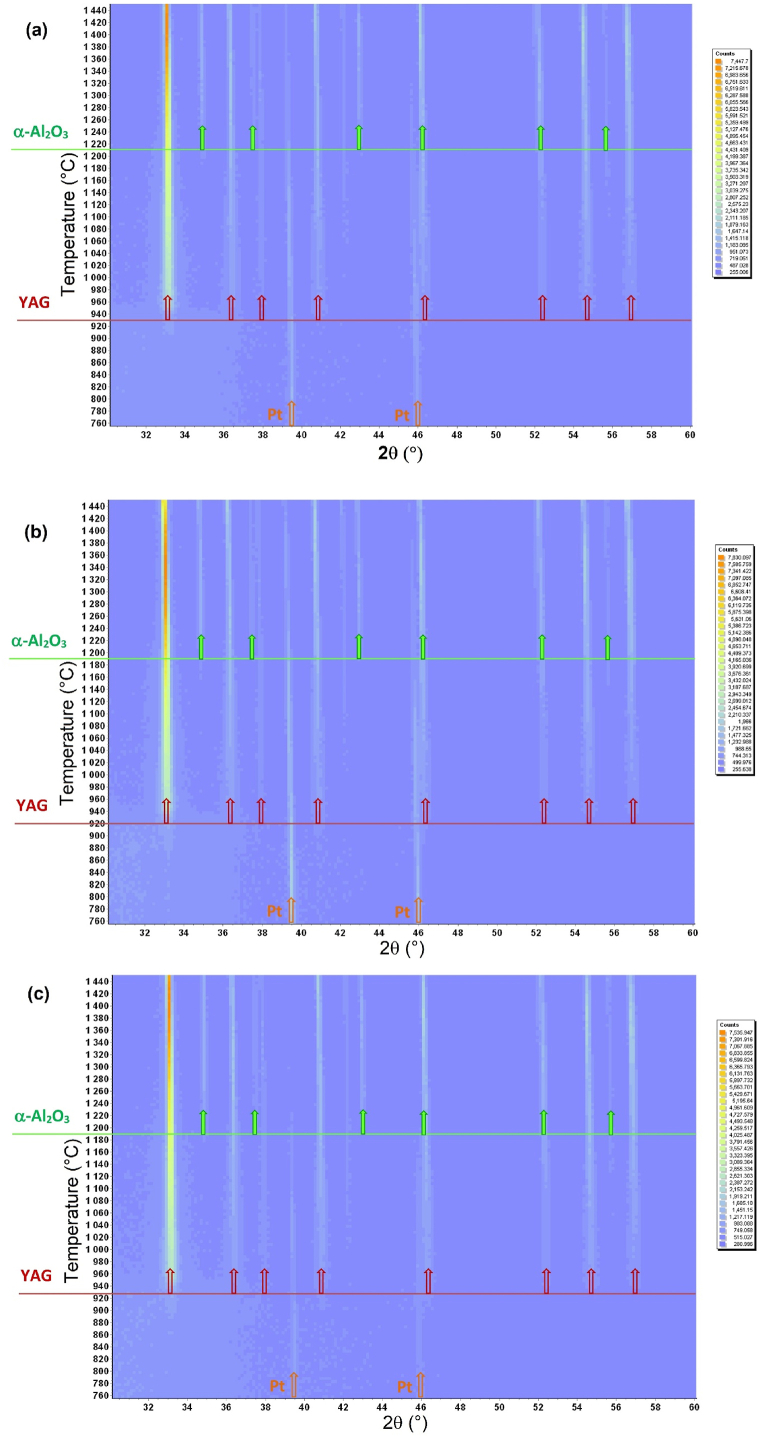
Heatmaps (temperature (°C) vs. position (^o^2θ) and the intensity is shown in colour) of the in-situ HT XRD data collected for the 6 h milled samples (a) S1; (b) S2; (c) S3.

The samples milled for 6 h in each experiment were also subjected to HT XRD analysis. In all XRD patterns obtained at the end of the HT XRD experiment (after cooling to 25 °C), the presence YAG phase and α-Al_2_O_3_ were documented. A comparison of short X-ray records is shown in Fig. 10. In all systems the crystallization of the YAG phase occurs first, in the temperature range from 920 to 930 °C. The onset of the crystallization of α-Al_2_O_3_ phase is in the temperature interval 1180–1210 °C. These comparisons show that the highest tendency to crystallization was observed in sample S2, where crystallization of the YAG phase started already at a temperature of 920 °C.

**Fig. 11:**
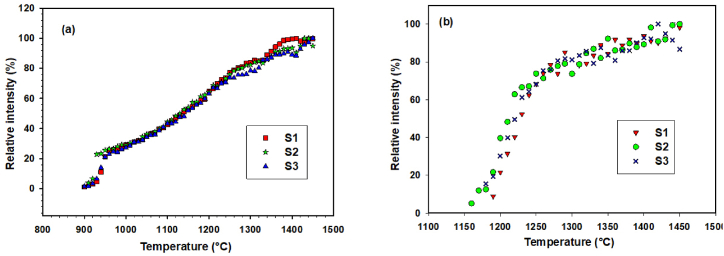
Temperature dependences of relative intensities of the <4.2.0> Y_3_Al_5_O_12_ diffraction line (a) and <1.0.4> α-Al_2_O_3_ diffraction line (b).

The intensities of the most intensive <4.2.0> YAG and <1.0.4> α-Al_2_O_3_ diffraction lines were evaluated, and the dependences of relative intensities on temperature were constructed (Fig. 11a, b). The highest increase of the relative intensity of the <4.2.0> Y_3_Al_5_O_12_ diffraction line was observed in the temperature interval 900–1300 °C. Above 1300 °C, the relative intensity of the YAG diffraction still increases, but the increase is insignificant.

For α-Al_2_O_3_, the onset of crystallization was observed in the temperature interval 1180–1200 °C. The highest increase of the relative intensity of the <1.0.4> α-Al_2_O_3_ diffraction line was observed in the temperature interval 1200–1300 °C. The temperature dependence of relative intensity of the most intensive peak <1.0.4> is in (Fig. 11b). The obtained dependences are very similar, and the applied milling conditions did not significantly affect the phase changes in the system.

### 3.3 Sinter ability

The samples milled for 6h from each series of milling were used to compare their sintering behavior. The samples were labeled as S1-HP, S2-HP, S3-HP. The conditions used in our previous HP experiments (T = 1600 °C, p = 30 MPa, and t = 0 min) were applied [16]. Dense, white samples with the density ranging from 4.313 to 4.235 g.cm^−3^ and relative density (RD) ranging from 96.4 % (sample S3-HP) to 98.2 % (sample S1-HP) were prepared. The value of RD was calculated as a ratio of the measured density of sintered samples and the theoretical density of fully crystalised material (4.39 g.cm^−3^). The measurement of mechanical properties revealed relatively high values of Vickers hardness (H_V_ = 16.2–17.6 GPa), which were comparable with the values achieved in our previous work [16,36]. Satisfactory values of the indentation fracture toughness (K_IC_) 4.3–4.6 MPa.m^1/2^ were also achieved. It is also interesting to compare the standard deviations of the determined properties, which could testify to the higher homogeneity of the S1 sample. The results are summarized in Table 6.

**Table 6:** Basic properties of sintered samples (T = 1600 °C, p = 30 MPa, and t = 0 min).

Sample	HP T/p/dwell time [°C/Pa/min]	16 [g.cm^−3^]	RD [%]	H_V_ [GPa]	K_IC_ [MPa.m^1/2^]
**S1-HP**	1600/30/0	4.315 ± 0.012	98.2	17.6 ± 0.2	4.3 ± 0.3
**S2-HP**	1600/30/0	4.235 ± 0.007	96.4	16.6 ± 0.9	4.5 ± 0.4
**S3-HP**	1600/30/0	4.313 ± 0.006	98.2	16.2 ± 0.7	4.5 ± 0.5

**Fig. 12:**
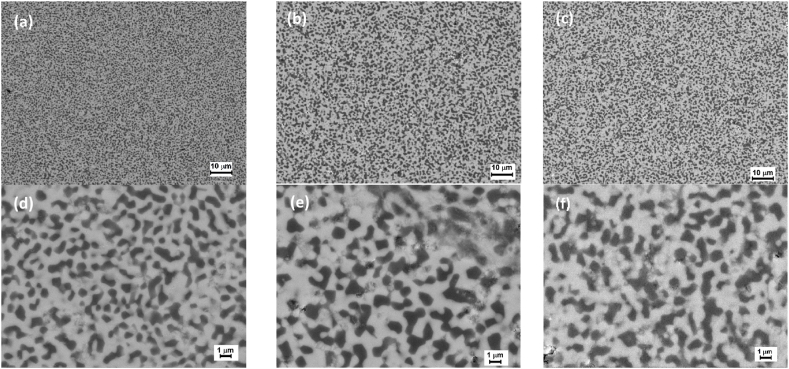
The SEM images of samples after HP experiments (a, d) S1-HP, (b, e) S2-HP and (c, f) S3-HP sample.

Detailed examination of the microstructure revealed a fine-grained microstructure composed of Al_2_O_3_ grains embedded in YAG matrix and an almost defect-free microstructure in sample S1-HP. The SEM images obtained at the same magnification (1000x and 5000x) are shown in (Fig. 12). Sintering of the sample S1 resulted in a fine-grained and uniform equilibrium eutectic structure with lamellar spacing (Fig. 12a, b). A similar structure was observed by Yasuda et al. [37]. In the remaining two samples, the structure was more coarse-grained, and clusters of small particles were observed (Fig. 12e,f). The size of the dark areas in the eutectic microstructures was determined with the use of the image analysis of the SEM micrographs.

**Fig. 13:**
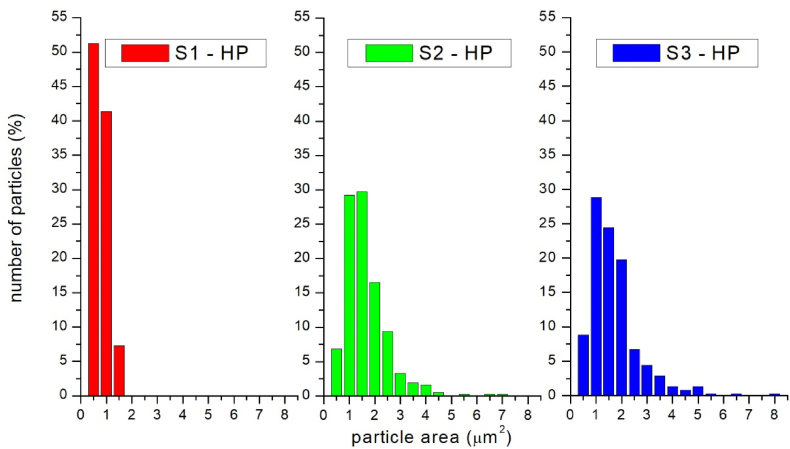
The results of grain size measurements in sintered samples.

Fig.13 shows the comparison of the image analysis results of sintered samples. The major fraction of sample S1-HP is created by dark particles with the areas ≤1 μm: their size usually does not exceed 2 μm. In the remaining two samples, the dark grain area in some cases was as large as 8 μm and composed of several connected grains. The highest fraction of grains was within the range ≤2.25 μm (87 % in sample S2-HP and 69 % in sample S3-HP). A comparison of samples S2-HP and S3-HP shows that sample S3-HP has the coarsest microstructure.

## 4 Discussion

The effect of particle size on thermal properties of YA glass microspheres with the eutectic composition (76.8 mol. % of Al_2_O_3_, 23.2 mol. % of Y_2_O_3_) was studied. The work was carried out to optimize the size of glassy particles for the subsequent HP sintering. To optimize particle size, three milling experiments were proposed. Results of PSA indicated sufficient grinding time of 6 h in all cases, while the milling experiments S1 and S2 appear to be the most suitable in terms of particle size and uniformity. In the first phase (1–3 h) of milling hollow microspheres were crushed. These hollow microspheres arise during flame synthesis due to the presence of residual carbon in the precursor powders and form a substantial fraction of particles with the size ≥20 μm [38]. Longer milling (4–9 h) probably only resulted in crushing of fragments of the hollow spheres into smaller parts, causing the fraction of particles with the size of 1–3 μm to increase and the distribution of particles to expand and becoming bimodal. The small particles can also form agglomerates with a size of up to 100 μm during further milling. The bimodal and trimodal distribution was observed in milling experiment S3. No fragments of solid spheres or battered spheres were observed in the SEM images after detailed inspection. This is attributed to the high hardness and abrasion resistance of studied glasses. By breaking the hollow particles, the probability of preparing highly homogeneous and defect-free microstructure increases, since hollow spheres can act as sources of defects (cavities and pores) during sintering. The controlled milling can thus improve the resulting microstructure of sintered materials.

Thermal analysis revealed the presence of one or two exothermic effects in the DTA curves, depending on milling time. Based on the results of our previous work [39] this can be attributed to crystallization of YAG phase in one or two steps. This assumption was confirmed by the results of XRD analysis of the samples after TA and HT XRD analysis milled for 6h. Only the presence of YAG phase was observed in the XRD patterns. The presence of γ-Al_2_O_3_ was not confirmed by XRD measurements of the samples after DTA in contrast with the YA microspheres after FS reported in Ref. [40]. This could indicate that crystallization of YAG in milled systems suppressed crystallization of intermediate alumina phase(s). The increase in the fraction of the YAG phase with increasing milling time in the samples after TA indicated an increasing tendency to crystallization in milled systems. This behavior can be attributed to several effects:

• heterogeneous crystallization prevails in the system, and milling gradually creates more crystallization centers in the sample,
• the surface area of the particles increases, thus promoting surface crystallization,
• homogeneous distribution of already existing crystallization centers and the centers created by milling, which supports crystallization in the entire volume of the sample.

Ultimately, after 6 h of milling, the system favors crystallization of the YAG phase in one step.

Sintering of the milled systems at 1600 °C, pressure 30 MPa and without holding time resulted in a formation of different microstructures. Sintering of particles milled by the regime S1 resulted in a very fine lamellar and almost defect-free eutectic microstructure with the grain size 1–3 μm. The powders prepared by milling regime S2 and S3 sintered to form a more coarse-grained microstructure. In terms of mechanical properties, satisfactory values of H_V_ (from 16.2 ± 0.7 to 17.6 ± 0.2 GPa) and indentation fracture resistance (from 4.3 ± 0.3 to 4.5 ± 0.5 MPa.m^1/2^), were comparable with those, reported in previous works [16,36]. The differences in size and size distribution of the powder particles after milling probably caused differences in the microstructure and mechanical properties of sintered bodies. The fine microstructure in the case of sample S1-HP can be attributed to the high uniformity of milled particles. Here, it can be assumed that there is a high degree of arrangement of seeds in the entire volume of the sintered sample. These seeds begin to crystallize almost immediately and, after a while, begin to hinder their growth. The result of this action is a finer microstructure. Also, an indication of bimodal particle size distribution (in the sample after 6h milling in the third experiment) and the associated presence of small ≤ 1 μm particles, which could create agglomerates in the sample, caused the formation of defects in the microstructure, which resulted in inhomogeneity of mechanical properties in the material. This is also documented by higher values of standard deviations of H_V_ and K_IC_ in samples S2-HP and S3-HP.

## 5 Conclusions

Yttrium-aluminate glasses with eutectic composition (76.8 mol % Al_2_O_3_ and 23.2 mol % Y_2_O_3_), and in the form of glass microspheres, were prepared by combination of the sol-gel Pechini metod and flame synthesis. Subsequently, three milling experiments were conducted to adjust the particle size and particle size distribution. The effect of grinding on thermal behavior, sintering ability of glassy microspheres, and on microstructure, density and mechanical properties of hot press sintered ceramic materials were thoroughly investigated. The prepared systems (raw and milled microspheres) were studied by PSA, DTA/TG, XRD, HT XRD and SEM. Only minor changes in temperature behavior and crystallization of pure YAG phase in one step were observed in all systems milled for 6 h. The sinter ability of the systems milled for 6 h was studied using HP sintering at the temperature of 1600 °C and pressure 30 MPa without isothermal dwell in inert atmosphere. It was shown that the milling conditions significantly affected the sintering ability, resulting microstructure and mechanical properties of the obtained materials. Dense (relative density; RV = 98.2 %) ceramic materials with a very fine lamellar eutectic microstructure, Vickers hardness of H_V_ = 17.6 ± 0.2 GPa and indentation fracture resistance K_IC_ = 4.3 ± 0.3 MPa.m^1/2^ were prepared from a powder milled at 200 rpm using the grinding bodies 5 mm in diameter. The results showed that the combination of flame synthesis and hot press sintering is, from a technical and economic point of view, suitable for the preparation of larger pieces of material for high-temperature applications and will also help to successfully implement this procedure in practice.

## CRediT authorship contribution statement

**Melinda Majerová:** Writing – original draft, Visualization, Investigation, Formal analysis, Data curation. **Anna Prnová:** Writing – original draft, Validation, Investigation, Data curation. **Jana Valúchová:** Visualization, Validation, Software. **Monika Michálková:** Visualization, Validation, Software. **Beáta Pecušová:** Investigation, Formal analysis, Data curation. **Michal Žitňan:** Investigation, Data curation. **Róbert Klement:** Validation, Data curation. **Dušan Galusek:** Writing – review & editing.

## Declaration of competing interest

The authors declare that they have no known competing financial interests or personal relationships that could have appeared to influence the work reported in this paper.
